# A high-content platform to characterise human induced pluripotent stem cell lines

**DOI:** 10.1016/j.ymeth.2015.11.012

**Published:** 2016-03-01

**Authors:** Andreas Leha, Nathalie Moens, Ruta Meleckyte, Oliver J. Culley, Mia K. Gervasio, Maximilian Kerz, Andreas Reimer, Stuart A. Cain, Ian Streeter, Amos Folarin, Oliver Stegle, Cay M. Kielty, HipSci Consortium, Richard Durbin, Fiona M. Watt, Davide Danovi

**Affiliations:** a HipSci Cell Phenotyping, Centre for Stem Cells and Regenerative Medicine, King’s College London, Great Maze Pond, London SE1 9RT, UK; b NIHR Biomedical Research Centre for Mental Health Informatics Core, King’s College London, De Crespigny Park, London SE5 8AF, UK; c Wellcome Trust Sanger Institute, Wellcome Trust Genome Campus, Hinxton, Cambridge CB10 1SD, UK; d European Molecular Biology Laboratory, European Bioinformatics Institute, Wellcome Trust Genome Campus, Hinxton, Cambridge CB10 1SA, UK; e Wellcome Trust Centre for Cell-Matrix Research, Faculty of Life Sciences, University of Manchester, Manchester M13 9PT, United Kingdom

**Keywords:** Cell based assays, High content, Phenotype screening, iPSCs, Induced pluripotent stem cells, Human pluripotent stem cells

## Abstract

Induced pluripotent stem cells (iPSCs) provide invaluable opportunities for future cell therapies as well as for studying human development, modelling diseases and discovering therapeutics. In order to realise the potential of iPSCs, it is crucial to comprehensively characterise cells generated from large cohorts of healthy and diseased individuals. The human iPSC initiative (HipSci) is assessing a large panel of cell lines to define cell phenotypes, dissect inter- and intra-line and donor variability and identify its key determinant components. Here we report the establishment of a high-content platform for phenotypic analysis of human iPSC lines. In the described assay, cells are dissociated and seeded as single cells onto 96-well plates coated with fibronectin at three different concentrations. This method allows assessment of cell number, proliferation, morphology and intercellular adhesion. Altogether, our strategy delivers robust quantification of phenotypic diversity within complex cell populations facilitating future identification of the genetic, biological and technical determinants of variance. Approaches such as the one described can be used to benchmark iPSCs from multiple donors and create novel platforms that can readily be tailored for disease modelling and drug discovery.

## Highlights

• iPSCs show inter/intra-line/donor-variability hampering characterisation.
• HipSci generates, banks and provides iPSCs from hundreds of individual donors.
• iPSCs respond to different human plasma fibronectin concentrations on 96-well assays.
• Phenotypic features: cell number, proliferation, morphology and intercellular adhesion.
• The methodologies described can be tailored for disease-modelling and other cell types.

## Graphical abstract

**Figure:**
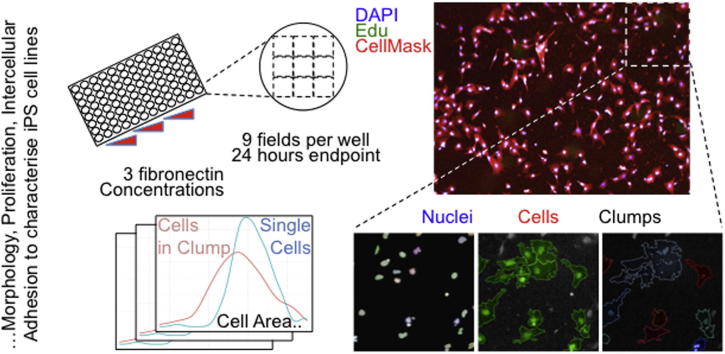

## 1 Introduction

Human induced pluripotent stem cells (iPSCs) offer tremendous potential not only for cell therapy but also to develop platforms for medical research. In particular, patient-derived iPSCs can be used to obtain selected differentiated cell types to model diseases and discover new therapeutics [1]. Heterogeneity in gene expression has been described within a specific iPSC line [2], between different donors [3], [4] and through the reprogramming process [5], [6]. Furthermore, several studies have focused on the differences between a small number of lines from patients and controls or used isogenic lines [7]. However, despite recent examples in this direction [8] dissecting the phenotypic heterogeneity within one cell line and among lines derived from the same donor or diverse individuals is yet to be fully explored.

Nonetheless, a clear definition of the genetic and epigenetic variance and how each of these affects cell behaviour in large panels of iPSCs is crucial for stem cell biology. Moreover, assessing the phenotypic variance observed in cell populations from multiple donors will facilitate scaling up culture systems as well as the development of quality control and automation protocols with undoubted value for the maintenance of pluripotent stem cells and controlled differentiation towards specific cell types.

The human induced pluripotent stem cells initiative (HipSci) is generating iPSCs from hundreds of healthy individuals as well as patients diagnosed with selected diseases. This represents a powerful resource to evaluate and quantify cell responses to chemical, physical and biological stimuli using novel assays and artificial microenvironments. Within this framework, phenotypic data are being collated with genomics, epigenomics and proteomics data to discover the impact of their variation on the cellular phenotype. Here we describe the development of a simple assay (including methods, workflow and set-up) to capture and quantify phenotypic features of iPSCs exposed to different extracellular matrix conditions.

## 2 Material and methods

### 2.1 iPSC quality control and maintenance

iPSCs are received from the Wellcome Trust Sanger Institute. There, cells are reprogrammed from fibroblasts using the Sendai virus method [9]. After reprogramming, each clone is genotyped and tested for copy number variations (CNVs). Pluripotency is assessed based on expression profiling [10], detection of pluripotency markers in culture and response to differentiation inducing conditions [11]. Data reported in this study refers to multiple replicate experiments of a single cell line [12] (Table 1, first line). iPSCs are passaged on Mitomycin-C inactivated mouse embryonic fibroblasts (MEFs) in Advanced DMEM/Ham’s F-12 supplemented with 20% v/v KnockOut Serum Replacement (all Life Technologies), 1% v/v l-Glutamine, 1% v/v Penicillin–Streptomycin (all Sigma–Aldrich), 55 mM 2-Mercaptoethanol (Life Technologies) and 4 ng/mL human bFGF (Millipore). Cells are split every 3–4 days using enzymatic and mechanical dissociation and media changed daily. Briefly, cells are washed with Dulbecco’s Phosphate Buffered Saline (DPBS, Sigma–Aldrich) and incubated with dispase and collagenase (all Life Technologies) for 10 min at 37 °C. The enzyme solution is then replaced with fresh culture medium and pluripotent colonies are dissected manually. Colonies are selected based on morphological features typical of human pluripotent stem cells and are split approximately 1:3 onto a new feeder plate.

CF-1 MEFs (Amsbio) are cultured in Advanced Dulbecco’s Modified Eagle Medium (DMEM)/Ham’s F-12 supplemented with 10% v/v Foetal Bovine Serum (all Life Technologies), 1% v/v l-Glutamine and 1% v/v Penicillin–Streptomycin (all Sigma–Aldrich). For iPSCs maintenance, inactivated MEFs are seeded as feeders on a 6-well microplate (Falcon) coated with 0.1% gelatin from porcine skin type A (Sigma–Aldrich) at a density of 10^6^ cells per 6-well plate and allowed to attach overnight.

### 2.2 Extracellular matrix coating conditions

To develop our assay, we first sought to identify favourable extracellular matrix substrates. We screened and examined a total of 74 diverse conditions from two sources (see Table 1). A customised array plate acquired from Orla protein technologies (Sarstedt cat. No. 02XECM-96) contained 9 conditions derived from single ECM proteins including fibronectin, laminin, collagen, vitronectin, osteopontin, tenascin C and bone sialoprotein and 17 conditions presented as a mixture of different ECMs. For this set, triplicates of a single concentration of approximately 25 μg/ml per condition were coated on wells. Additionally, we created an array plate containing in duplicate fragments of fibrillin-1, fibrillin-2 and agrin as well as cellular and plasma fibronectin at a range of 1, 10 and 25 μg/ml. The plate also contained single concentrations of the following ECM proteins: LTBP-1 C-terminal fragment, MAGP-1, syndecan-2 extracellular domain, syndecan-4 extracellular domain and fibulin 4. Extracellular matrix proteins diluted in 80 μl PBS were incubated overnight on 96 well μClear black tissue culture plates (Greiner cat. No. 655090) at 4 °C. The supernatant was removed and well-coating blocked by the addition of 10 mg/ml BSA for 1 h. Upon removal of the BSA solution, the plates were stored at −80 °C prior to use. Bovine Serum Albumin (BSA) and uncoated tissue culture plastic (TCP) were used as controls.

For the fibronectin assay, 96-well μClear plates (Greiner) are coated with 1, 5 and 25 μg/ml human plasma fibronectin (Corning) and stored at 4 °C (overnight or up to 14 days). We will refer to these conditions as Fn1, Fn5 and Fn25, respectively (whereas Fn10 was only used in the screening). Each is present in a technical triplicate on the same vessel randomised per column using diverse patterns (*i.e.* Fn1-Fn5-Fn25, Fn1-Fn25-Fn5, Fn5-Fn25-Fn1, Fn5-Fn1-Fn25, Fn25-Fn1-Fn5, Fn25-Fn5-Fn1). Border wells are avoided to reduce edge effects. Before use, fibronectin is removed and wells are washed with DPBS (Sigma–Aldrich).

### 2.3 Assay set-up: cell seeding, fixation, staining and image acquisition

When iPSCs cultures reach approximately 80% confluency, cells are washed with DPBS and dissociated with collagenase and dispase for 45 min at 37 °C. Pluripotent colonies detach from the microplate surface and are further dissociated with Accutase (Innovative Cell Technologies) for 5 min at 37 °C. The single-cell suspension is centrifuged for 3 min at 400 rpm after which the supernatant is removed and cells re-suspended in fresh culture medium (Section 2.1) supplemented with 10 μM Y-27632 Rho-associated protein kinase (ROCK) inhibitor (Enzo Life Sciences). Cells are then counted using a Scepter 2.0 automated cell-counting device (Millipore) and seeded onto the fibronectin-coated 96-well plate using Viaflo (INTEGRA Biosciences) electronic pipettes.

At 23.5 h after seeding cells are labelled with EdU (Click-iT EdU kit, Life Technologies) for 30 min. For fixation, 8% paraformaldehyde (PFA, Sigma–Aldrich) is added to an equal volume of medium for a final concentration of 4%, and left at room temperature for 15 min. After fixation, cells are washed with DPBS (Sigma–Aldrich) and stored at 4 °C. Cells are then blocked and permeabilised with 0.1% v/v Triton X-100 (Sigma–Aldrich), 1% w/v bovine serum albumin (BSA, Sigma–Aldrich) and 3% v/v donkey serum (Sigma–Aldrich) for 20 min at room temperature. After washing with DPBS, cells are stained with Click-iT EdU kit (Life Technologies) according to the manufacturer’s instructions except the azidofluoride reaction buffer halved in DPBS. After 1 h cells are washed with DPBS, stained for 1 h at room temperature with CellMask plasma membrane stain (1:1000, Life Technologies) and DAPI nuclear stain (1:5000, 1 μg/ml final concentration, Life Technologies). Plates are then washed with DPBS and stored at 4 °C. EdU was used according to manufacturer’s instructions except for the concentration of the azide reagent halved. A period of half hour was chosen in line with the cell cycle period described in the literature for human iPSCs [6]. As a control, cells were exposed to the same reagents in the absence of EdU incorporation showed comparable background intensity values to the cells considered EdU negative by our analysis. Acquisition parameters and image analysis pipeline are described in details in Section 3.1 and Section 3.3 respectively. For endpoint analysis, stained plates are imaged using an Operetta® (Perkin Elmer) high content device. Images are acquired in wide field mode using 4 channels (DAPI, 488, 647, Brightfield as control). On Greiner μClear plates, we optimised heights focal settings for brightfield, DAPI, EdU and CellMask (respectively 11, 20, 9 and 10 μm) following the sharpest focal plan guided by the highest intensity of signal. Times of exposure (respectively 100, 200, 300 and 10 millisecs) were chosen to minimise the time of acquisition and the amount of reagents used. Incucyte (Essen Bioscience) images were acquired largely as described in [13].

## 3 Results and discussion

We first aimed to obtain a robust read out to evaluate response of undifferentiated iPSCs to controlled changes in the microenvironment. Furthermore, we aimed to develop a set of procedures to effectively extract from images relevant phenotypic features, which can be quantified and interrogated in downstream phases of the analysis. As a proof of principle for this protocol, we used here dissociated iPSCs from a single control line in undifferentiated culture conditions. This study serves as a foundation to build phenotypic signatures of large panels of iPSC lines from multiple donors which can be collated to complementary and matched datasets containing genomic and proteomic information. Similar approaches can be readily tailored to study cells differentiated from pluripotent stem cells or generated by other reprogramming strategies.

### 3.1 Screening for optimal extracellular matrix protein conditions

Cell behaviour is heavily influenced by genetics and by the surrounding environment [14], [15]. In order to evaluate specific differences on cell behaviour, we reasoned that diverse coating concentrations on multiwell plates could be exploited. Thus, as a prerequisite to build a scalable workflow suitable for the characterisation of large panels of iPSCs, we first set out to identify an effective, robust and inexpensive substrate. We searched for an extracellular matrix (ECM) protein or peptide that could be used at different concentrations ranging from unfavourable to permissive for cell attachment and cell spreading. Furthermore, we searched for conditions to robustly detect a sufficient number of single cells when plating the same number of iPSCs on a range of diverse concentrations. In addition to these criteria, we also aimed to keep the concentrations as low as possible to minimise the presence of potential contaminants (*e.g.*: growth factors).

We tested 74 conditions (described in Section 2.2 and Table 1) by seeding cells, imaging them live every hour for 24 h and fixing and staining with DAPI, EdU and CellMask. Several conditions such as fragments lacking RGD sites (*e.g*. from Agrin, Syndecan, Fibulin 4 and MAGP-1) yielded poor numbers of cells, similar to the BSA or tissue culture plastic controls (Table 1, left insert). Others, such as vitronectin allowed for attachment, spreading and survival and yet cells were rarely found as single cells appearing mostly in clumps (Table 1, right insert). Importantly, some of the conditions tested allowed the attachment, spreading and survival of single cells and additionally demonstrated a dose-dependent response. These results suggest that varying concentrations of one single substrate may lead to the establishment of assays tuning cell response in terms of attachment, spreading, proliferation and intercellular adhesion. For our screening experiments we used two different vessel types: Sarstedt 96w (for the Orla plate) and Greiner μClear (for the custom plate). Images acquired on the Operetta appeared sharper for the Greiner μClear. Objective 4×, 20× and 40× were unpractical or gave an unfavourable ratio of cells on the borders versus cells in the field. We therefore used a 10× long working distance (WD) objective for 9 fields of view per each well, excluding the peripheral fields and excitation 50% and transmission 50% according to manufacturer’s instructions to avoid photo-bleaching.

### 3.2 Assay development using a gradient of fibronectin concentrations

Among all promising substrates (Table 1, #65–74), we focused on those that for practical reasons such as cost and robustness will result suitable to a large number of iPSCs. Furthermore, these might facilitate the scale up and industrial application of similar strategies. Fibronectin is a large glycoprotein generally in the form of an insoluble dimer. Several reports suggest an essential role for fibronectin during vertebrate embryonic development [16] and tissue regeneration [17]. In human iPSCs, several studies indicate that fibronectin is a permissive substrate for the maintenance of pluripotency [18], [19], [20], [21], [22], [23]. In addition, its adsorption on tissue culture plates has been shown to give rise to diverse structures with diverse surface density affecting the number of focal adhesion contacts [24]. In the human body, fibronectin exists in two forms: the plasma form circulates in the blood whereas cellular fibronectin is physically associated to the cell surface [25]. Both cellular and plasma fibronectin as well as other conditions presenting recombinant peptides gave similar results in terms of total number of cells attached and number of single cells. Fn1 and Fn25 conditions yielded remarkable differences indicating an environment conducive of low versus high cellular adhesion respectively, whereas Fn10 appeared very similar to Fn25. We therefore sourced plasma fibronectin in conditions Fn1-Fn5-Fn25 to validate our observations. To achieve statistical significance, these conditions were each replicated three times on the same vessel in a randomised pattern (Fig.3A). We report here results from numerous replicate experiments (*n* = 41).

**Fig. 2:**
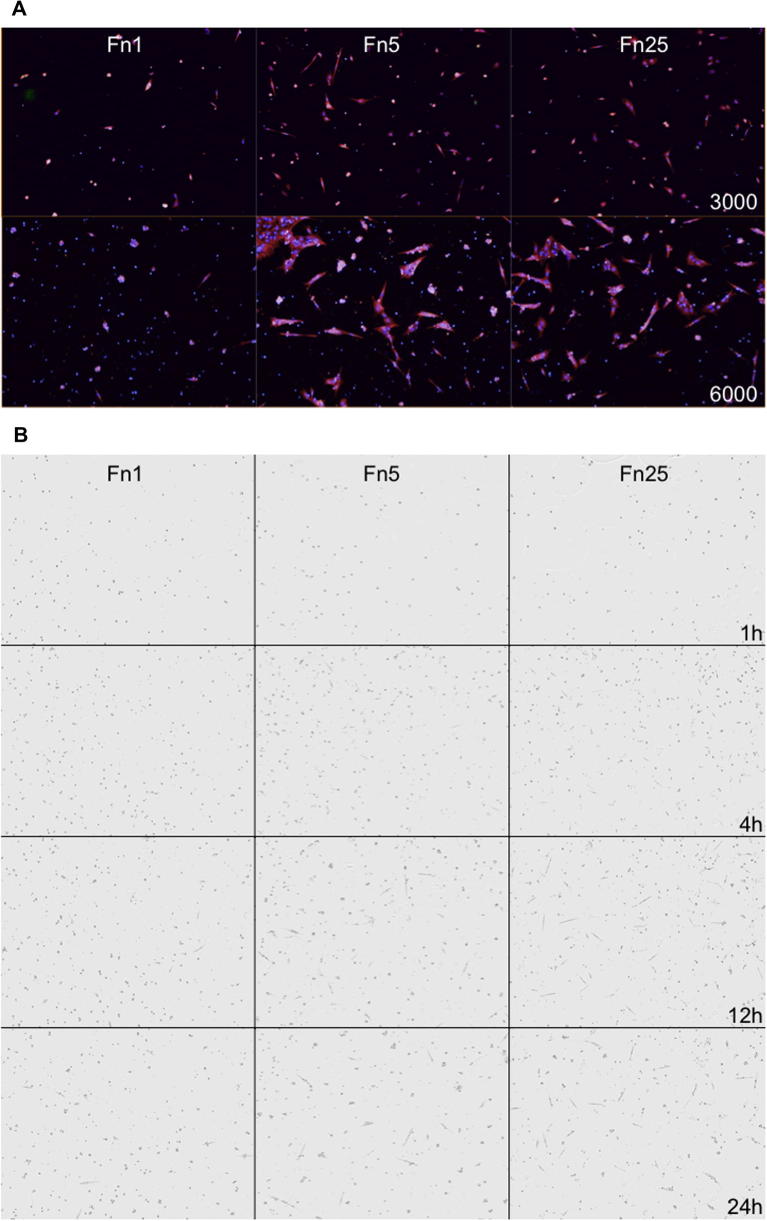
Assay development for cell density and time before fixation. (A) The panels show representative microphotographs for 3000 (Top) or 6000 (bottom) cells plated on different fibronectin concentrations (Fn1, Fn5, Fn25). Note that the majority of cells when 6000 cells are plated appear in clumps. Blue, DAPI. Green, EdU. Red, CellMask. One field of view per well is shown here. (B) Live image movies were derived and inspected of cells plated as 3000 cells on the three fibronectin conditions. Timepoints 1 h, 4 h, 12 h and 24 h after seeding are shown here. Adhering cells can be readily observed from 4 h onwards and spreading has occurred in most cells by 24 h.

Having chosen the extracellular matrix and a suitable range of concentrations, we tested the initial cell density. A seeding density below 3000 cells per well resulted in a suboptimal number of single cells. On the other hand, when seeding 6000 cells on Fn25 we observed a comparable number of single cells to those observed when plating 3000 cells on Fn25. We also observed a high number of clumps forming when plating 6000 cells on Fn1 (Fig.2A). We thus chose to plate 3000 cells per well as this seeding density yielded an optimal number of single cells in all conditions tested.

To determine the most appropriate duration for our endpoint assays we performed live imaging of cells plated in Fn1, Fn5 or Fn25. The time chosen should be long enough for the cells to adhere and be in line with the cell cycle described for human iPSCs [26] to minimise complete cytokinesis. Ideally in this condition, the total number of cells observed is deemed to depend more on cell adherence and survival rather than on cell division. DNA replication can then be validly assessed with a 30 min pulse of EdU. Cells appeared to start adhering and spreading around 4 h post-plating at all fibronectin concentrations. At 24 h, the vast majority of the attached cells will have completed their spreading with minor cell divisions observed (Fig.2B). We therefore opted to run the endpoint assays 24 h after plating.

### 3.3 Image analysis pipeline

**Fig. 3:**
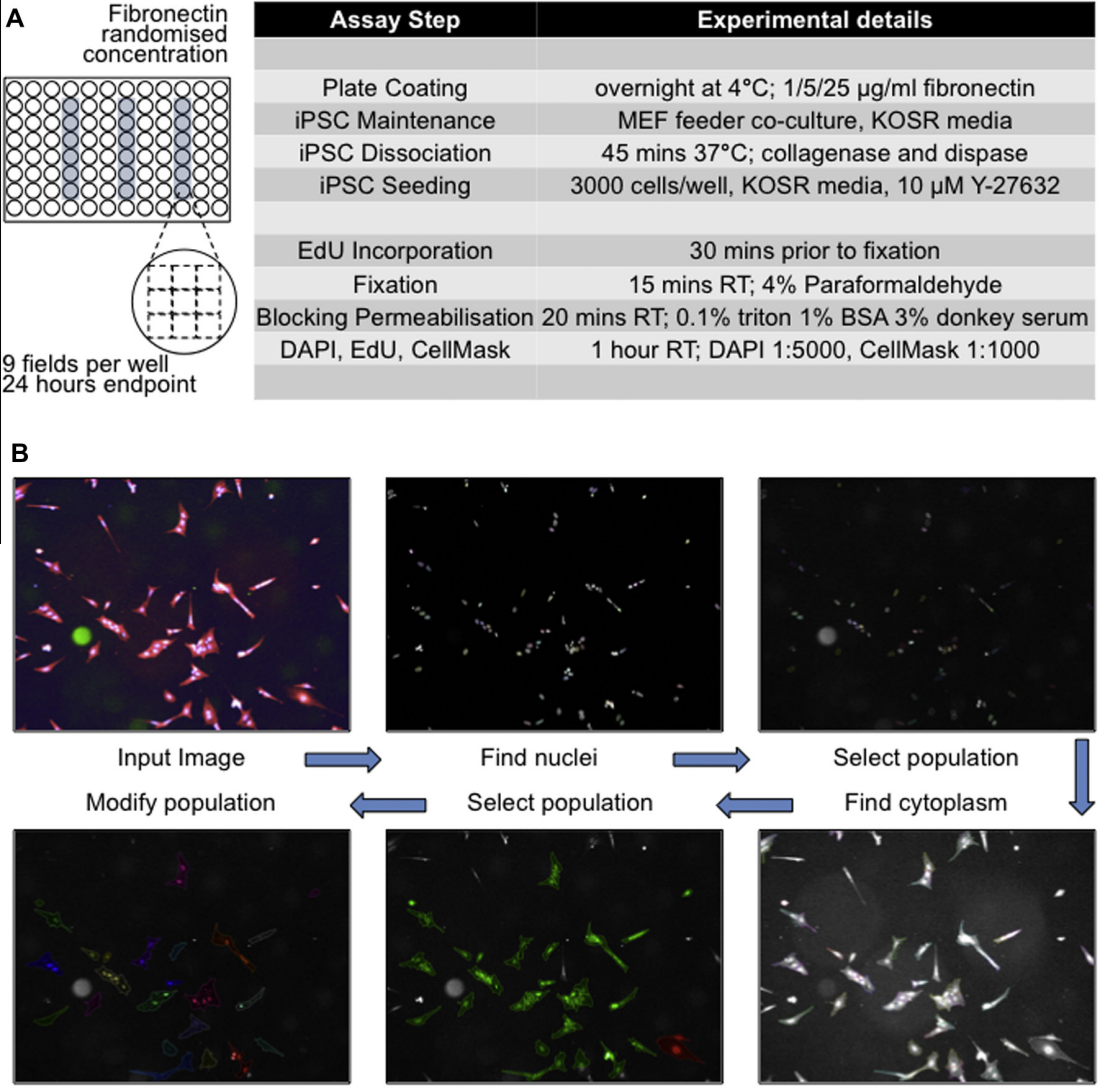
Workflow diagram detailing image acquisition and analysis. (A) Layout of the 96-well plates for phenotype assays showing fibronectin concentration per column (blue). Different experiments present randomised patterns (*i.e.* Fn1-Fn5-Fn25, Fn1-Fn25-Fn5, Fn5-Fn25-Fn1, Fn5-Fn1-Fn25, Fn25-Fn1-Fn5, Fn25-Fn5-Fn1). Step-by-step experimental conditions for the assay set-up are detailed on the right. KOSR = KnockOut Serum Replacement, RT = room temperature, BSA = bovine serum albumin. (B) Image analysis pipeline detailed in Section 3.3 is summarised here. Input images are segmented to identify nuclei and cytoplasm. Border objects and artefacts are discarded via morphology and intensity assessment on nuclei and on cells. The modify population module is employed to identify clumps based on cell-to-cell proximity and capture the number of cells in each clump as a context feature.

Image acquisition parameters using Operetta have been detailed in Sections 2, 3. We here describe in details the image analysis pipeline we have built using the Harmony 3.5.2 software (summarised in the workflow diagram in Fig.3B). Similar strategies could be easily transferred to other, possibly open-source, image analysis software platforms.

**Fig. 4:**
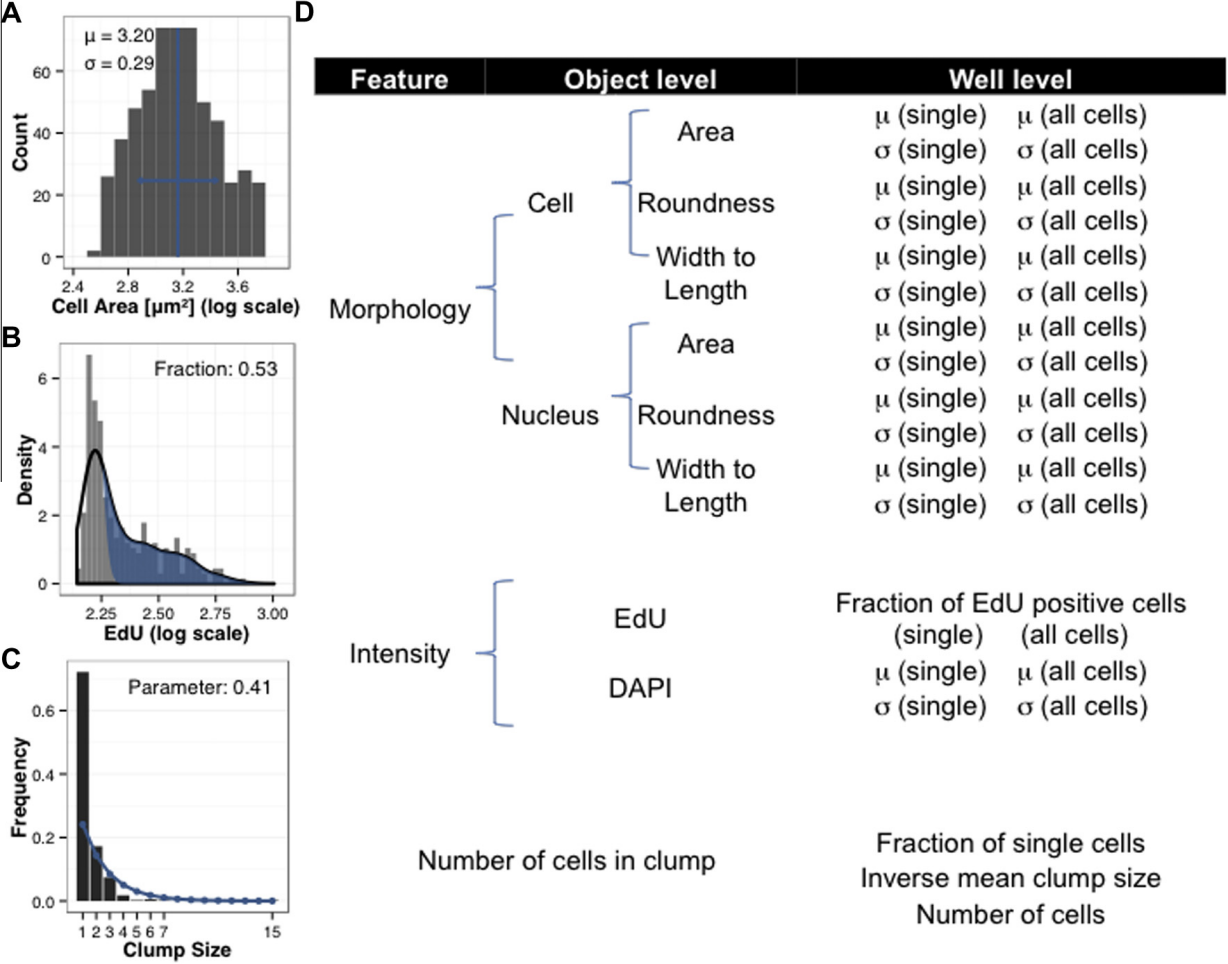
Phenotypic features analysed and their aggregation. Cell-based measurements are aggregated in each well to well-based measurements. (A) Histogram of cell areas with highlighted sample mean and standard deviation (in blue). (B) The EdU median intensity is aggregated using a Gaussian distribution fitting to the main peak of cells, which is considered the EdU negative cells population. The area under the curve that is not explained by that main peak is considered the fraction of EdU positive cells (in blue). (C) Distribution of clump sizes in well and the fit of a geometric distribution (in blue). Clump size refers to the number of cells in a clump. The inverse mean is the parameter of that fit and is used to describe the tendency of the cells to form clumps. (D) Shows detail of all phenotypic features for single cells and all cells. μ = mean σ = standard deviation.

We first used the Input Image module and proceeded to minimise background by processing individual planes with a basic flatfield correction and no quick tune. The find nuclei module segments nuclei using the DAPI channel and the M Method. A 18 μm diameter was chosen with 0.40 splitting coefficient and 0.05 common threshold. These parameters allowed to efficiently segment an output population named ‘Nuclei’ still carrying artefacts to be discarded based on sizes and intensity of signals. Within the calculate morphology properties module we used the standard methods on the nuclei population and on the nucleus region to define area, roundness and ratio width to length and output properties as ‘Nucleus’. We then used calculate intensity properties modules for the Alexa488 channel (EdU), the brightfield and the DAPI outputting median intensities as properties for each object. Using the select population module we then filtered by properties as follows: nucleus area between 60 μm^2^ and 600 μm^2^, EdU median intensity below 10.000 and DAPI median intensity between 500 and 10.000 with brightfield over 0. This refined output population was named ‘Nuclei 2’. We then used the find cytoplasm module on channel CellMask Deepred on nuclei 2 choosing method A with threshold 0.05. We select population again for ‘Nuclei 2’ removing border objects (common filters) on cell region. The output population was named ‘Cell unselected’. We then calculate morphology properties for cell unselected on cell using standard method and area, roundness and ratio width to length. The output properties were named ‘Cell’. We then applied the select population module using ‘Cell unselected’ and filter by property cells with cell area below 6000 μm^2^. Thus, filters are applied discarding objects of nucleus area below 60 μm^2^ and over 600 μm^2^, EdU median intensity over 10,000 AU and DAPI median intensity under 500 or over 10,000 AU and cell area bigger than 6000 μm^2^ (the latter include the vast majority of the few feeder cells present on the wells, see Fig.3B). From the modify population module we used the cluster by distance method on the ‘cell’ population on cell region. Distance was set as 0 and Area over 0 px and no fill holes. The output population was named ‘Clumps + Singles’. We then used the calculate properties module on the ‘Clumps + Singles’ population choosing the by related population method. Related population cell and number of cell was considered as a property. The output properties were defined ‘per clump’. We then applied the calculate properties module to the cell population using by related population and using the number of cell per clump leaving the output property blank. In practical terms, a population is here modified to conglomerate ‘cells’ in a related ‘clump’ and to assess the number of ‘cells’ in each ‘clump’ and tag this back from the related population into each ‘cell’ object having this value equal one for single cells. We adapted this strategy from similar approaches [27]. We then used the define results module exporting for the ‘cell’ population only the following parameters for all cells producing single cell results as selected. In total, for each ‘cell’ object, 9 phenotypic features are defined: 6 morphology, 2 intensity and 1 context feature. The morphology features selected are: nucleus area, nucleus roundness, nucleus width to length ratio, cell area, cell roundness, cell width to length ratio; The intensity features are: DAPI median and EdU (488) median and the context feature captured is: Number of cell per clump sum (see Fig. 4).

### 3.4 Phenotypic features and their aggregation

For structured access, the output of the Harmony image analysis pipeline was stored in a MySQL database along with experimental metadata including fibronectin concentration per well and experiment number. Further processing, analysis and data visualisation was performed within the statistical computing framework *R* directly accessing the database.

The cell phenotypic features described above were suitably normalised in value (log10 or square transformation) and aggregated across the cells for each well by taking average and standard deviation (Fig.4A). The cell number was directly acquired from the Harmony data. For EdU, median intensity raw values were grouped and characterised on a well-based measure by the fraction of positive cells. We opted to quantify this as the area under the empirical density not explained by a Gaussian main peak representing EdU negative cells (see Fig.4B). Tendency of cells to form clumps was described for each well by two summary statistics: the fraction of single cells and the inverse of the mean clump size. From the distribution of clumps over clump sizes (Fig.4C) we observed exponentially less clumps of bigger sizes. The inverse of the mean clump size as the defining parameter of that geometric distribution proves therefore valid. Additionally, wells with fewer cells overall tend to have more single cells in proportion, as the chance of cells to come to contact is lower. Percentage of single cells was also used as a measure of the ability of cells to form clumps.

**Fig. 5:**
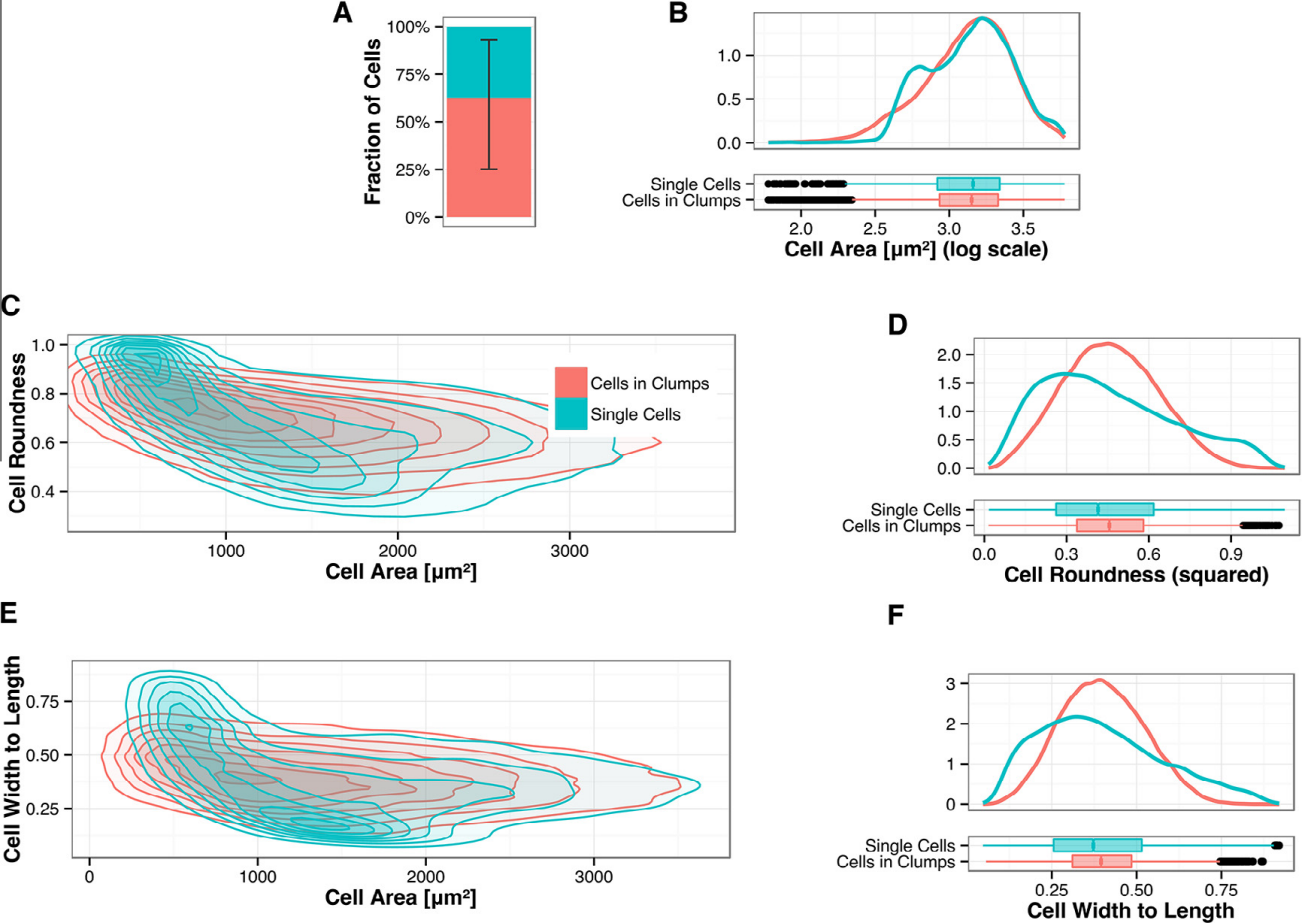
Phenotypic features vary in single cells and cells in clumps. Values of phenotypic features for single cells (blue) and cells in clumps (red) surrounded by at least one neighbouring cell, examined across the fibronectin concentrations in a series of replicate experiments. (A) Shows the fraction of single cells obtained by the assay. Over one third of cells (37%) appear as single cells. Min–Max range is shown. (B–F) morphological cell features for single cells and cells in clumps. Cell area, roundness and cell width to length display a wider range in single cells versus cells in clumps. This is suggestive of constraint from neighbouring cells limiting the shape of cells in clumps. (D) Cell roundness and (E) cell width to length are plotted versus cell area. These data are shown as examples of the validity of this method to capture features emerging upon cell–cell contact.

We used the Number of cell per clump sum as a context feature to maintain all objects in a common database interrogating separately data from single cells versus from cells in clumps (Fig. 5). Our assay demonstrated over 41 replicates a fraction of single cells per well of 37% indicating the effectiveness of the chosen substrate conditions in preserving a population of single cells (Fig.5A). Exploiting this strategy, we first asked whether cell–cell contact affects phenotypic features. Cell area appeared different depending on the context feature and showed a bimodal curve in single cells (Fig.5B, C, E) indicating the presence of a population of smaller single cells likely not spreading. We postulated that cells that come in contact with neighbouring cells may show distinct morphology from single cells as they are constrained in shape by the presence of their neighbours and cannot elongate as single cells. In fact, cell roundness (Fig.5C, D) and cell width to length (Fig.5E, F) showed a wider range for single cells than for cells in clump. These results indicate that our assay is suited to capture differences in features emerging upon intercellular adhesion. We next sought to observe and quantify the effect on phenotypic features of different fibronectin concentrations.

### 3.5 Diverse fibronectin concentrations trigger specific phenotypic responses

**Fig. 6:**
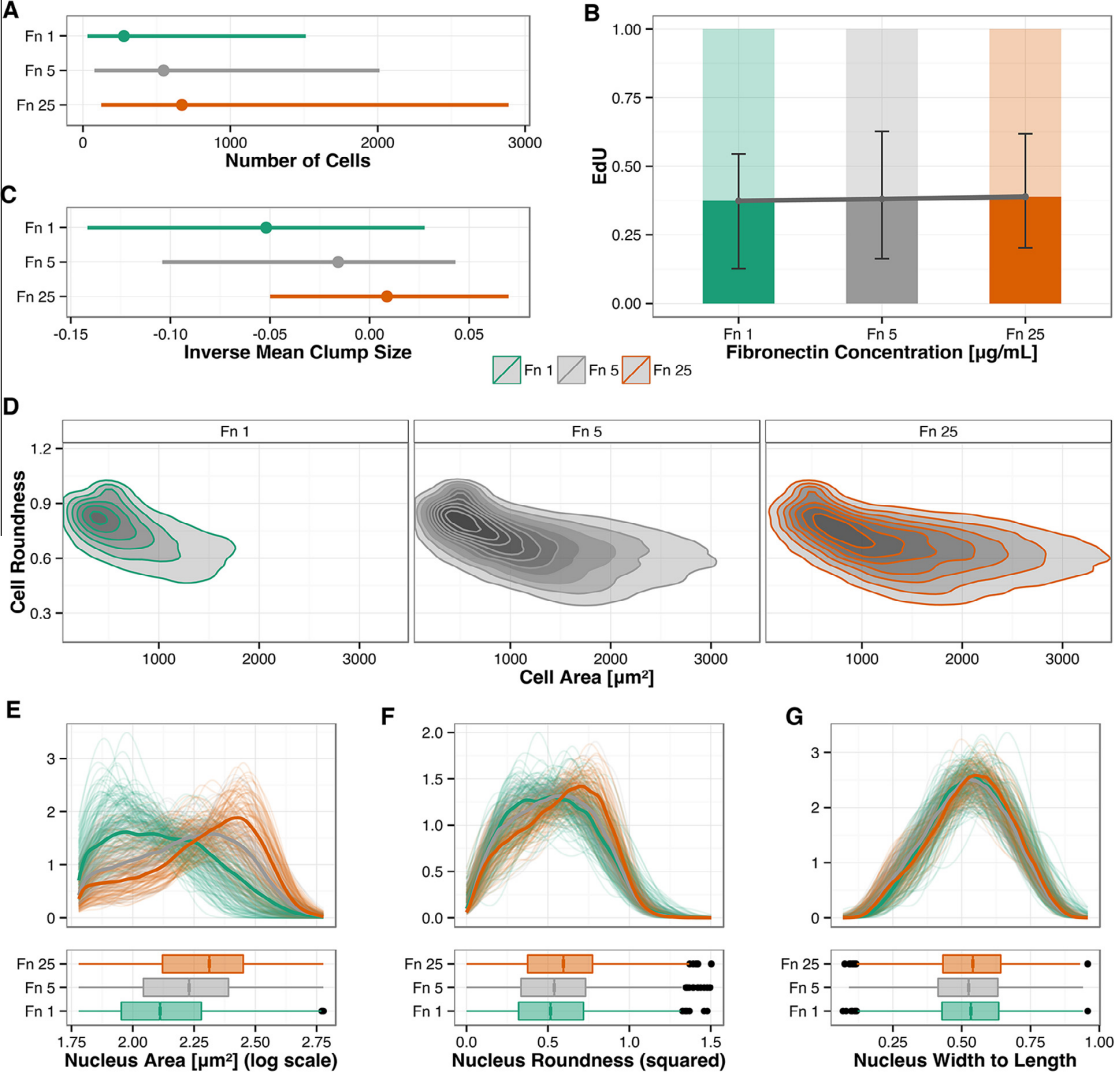
Fibronectin conditions affect several phenotypic features. Phenotypic features were interrogated in a series of replicate experiments for each of three fibronectin concentrations in all cells (Fn1; green, Fn5; grey, Fn25, red). (A) Number of cells per well. Min–Max range is shown. (B) Fraction of EdU positive cells over the three fibronectin concentrations. Min–Max range is shown. (C) Inverse mean clump size, an indicator of the propensity of cells to clump. (D) Cell roundness plotted over cell area. Note that cells in higher concentration of fibronectin present a wider range of values. (E–G) Density plots for nucleus area, nucleus roundness and nucleus width to length. The *y* axe shows the relative fraction of cells. Min–Max range is shown. In all graphs: green, Fn1; grey, Fn5; Orange, Fn25.

We therefore asked whether changes in the concentration of fibronectin affected the number of cells on the plate. We observed as expected that the total number of cells was higher in Fn25, intermediate in Fn5 and lower in Fn1 (Fig.6A). We are seeding the same number of cells and the period of observation is relatively short and consistent with the time required to attach and spread before a cycle (see Section 3.2). Thus, we speculate that this difference in number of cells retrieved is more likely due to differences in adherence and/or survival and less likely due to proliferation. Accordingly, the fraction of EdU positive cells was comparable (Fig.6B). Also, we found an increased tendency to form clumps in Fn25 in terms of inverse of mean clump size (Fig.6C). We cannot rule out that this may be an indirect effect of fibronectin through cell number or cell migration. In agreement with visual inspection, both cell and nuclear morphology varied substantially in these diverse conditions. We found wider ranges in cell area on Fn25 whereas cells appeared more round on Fn1 (Fig.6C). Similar observations were made for width to length (not shown). Density plots of nuclear area (Fig.6D), roundness (Fig.6E) and width to length (Fig.6E) also presented differences in these features at a population level.

**Fig. 7:**
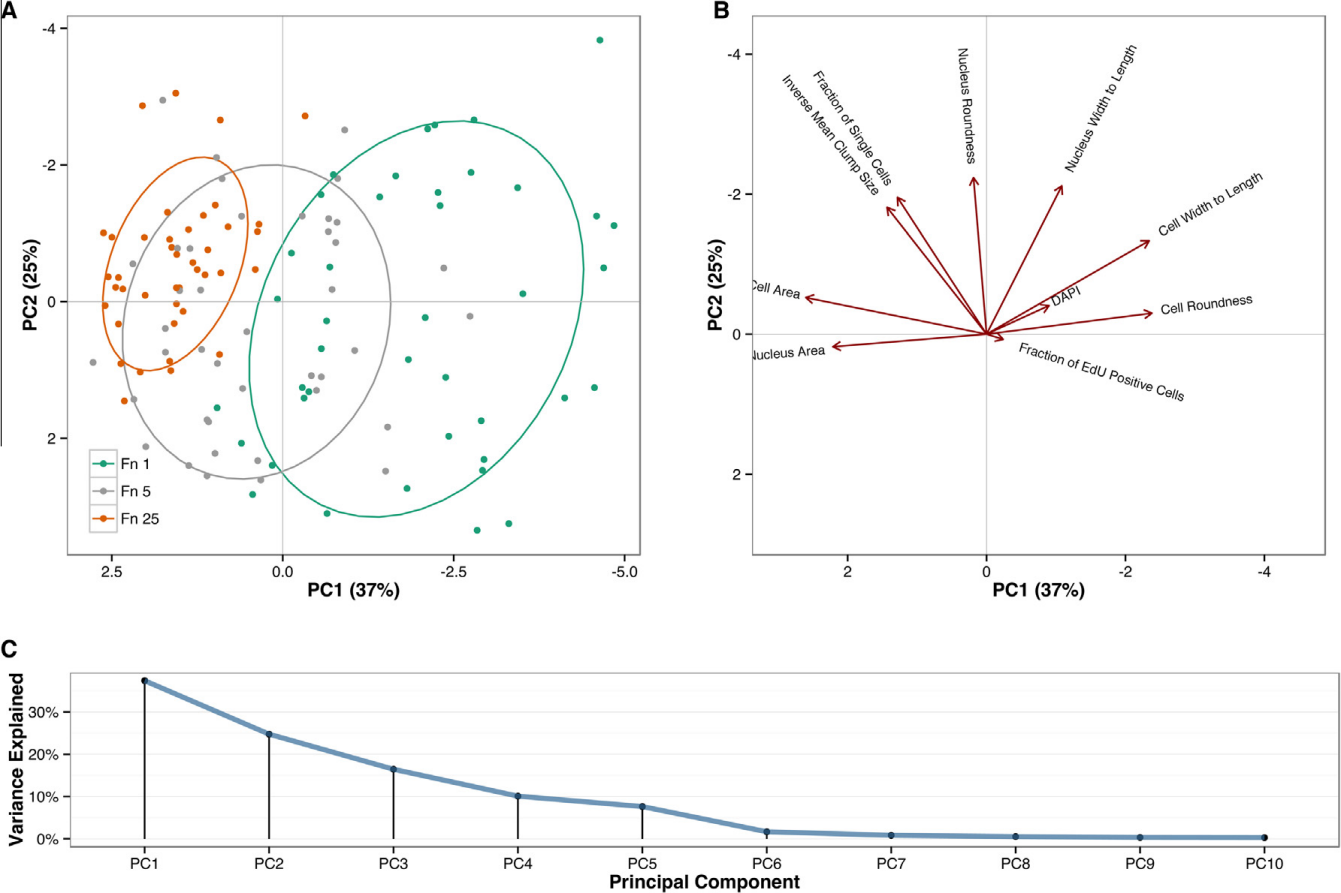
Fibronectin conditions mediate distinct phenotypic responses. (A) Principal component analysis is performed on the described phenotypic features in a series of replicate experiments. Green, Fn1; Grey Fn5; Orange Fn25. The ellipses represent the higher dimensional space defined by 68% of samples. (B) Directionality of the contributions of each phenotypic features to the first two components. (C) Percentage of variance explained by each component. Phenotypic features extracted from cells exposed to different fibronectin concentrations segregate in a high dimensional space.

We finally asked whether the developed assay and the cell phenotypic features obtained were sufficient to separate in a high dimensional features space cells in different fibronectin concentrations. We thus performed principal component analysis (PCA, Fig.7A). The defined features affected variably the two components (Fig.7B) which together explained a percentage of variance of approximately two thirds (Fig.7C). An elliptic area representing 68% of the sample space showed non-overlapping Fn1 and Fn25 conditions whereas Fn5 appeared intermediate. Altogether these results demonstrate that the high content platform we developed is suited to quantify phenotypic feature changes in human iPSCs that depend on cell–cell contact and biological responses triggered by different substrate conditions.

### 3.6 Potential modifications to the current pipeline and conclusions

We deliberately chose a simple cytochemistry based read-out using only dyes to set up our workflow. Nonetheless, the content of this assay read-out can be easily increased using antibodies and other reporters. We have observed that the binding to fibronectin is partially blocked by disturbance with an anti-β1 integrin antibody indicating a specific effect of the substrate on the cell surface iPSCs. Blocking antibodies, inhibitors as well as other different environments could be used in similar approaches to challenge cell responses. The methods were developed on feeder-dependent iPSC lines and also successfully tested on feeder free cells. One possible confounding factor is the presence of sparse feeder cells among the iPSCs. Reassuringly, and as a negative control, dissociation with dispase and collagenase did not dissociate a cell culture composed of feeder cells only (Data not shown). The rare contaminating feeder cells are in the vast majority discarded based on their size. Changes in the pipeline such as the introduction of a machine learning classifiers were therefore deemed not necessary for this level of contamination but may be considered in the future for similar studies. It is also possible to exclude clumps over a certain size to contain artefact from suboptimal seeding although this was also deemed not necessary with the current experimental conditions. To enrich the panel of morphological features analysed to train classifiers, an extensive array of other morphological features could also be derived in combination with the described morphology features. Specific phenotypic traits can be examined further, for example improving the adherence would result in larger production of cells in a faster, more efficient and cost-effective manner. Furthermore, controlling the distribution of single cells versus clump may help the development of methods for homogeneous delivery to the cells of factors and a more stringent control of differentiation protocols. In conclusion, the characterisation of large panels of iPSCs is an important and challenging task. The method we describe here can be applied to test large panel of iPSCs to benchmark and characterise their phenotype and can readily be tailored to the acquisition of other parameters and the analysis of differentiated cell types.

## Funding bodies

Maximilian Kerz and Amos Folarin are funded by the National Institute for Health and Research Biomedical Research Centre at South London and Maudsley NHS Foundation Trust and King’s College London. Stuart Cain is funded by the United Kingdom Regenerative Medicine Platform. HipSci (www.hipsci.org) is funded by a Grant from the Wellcome Trust and the Medical Research Council.

## Conflict of interest

No conflict.

## Figures and Tables

**Fig. 1:**
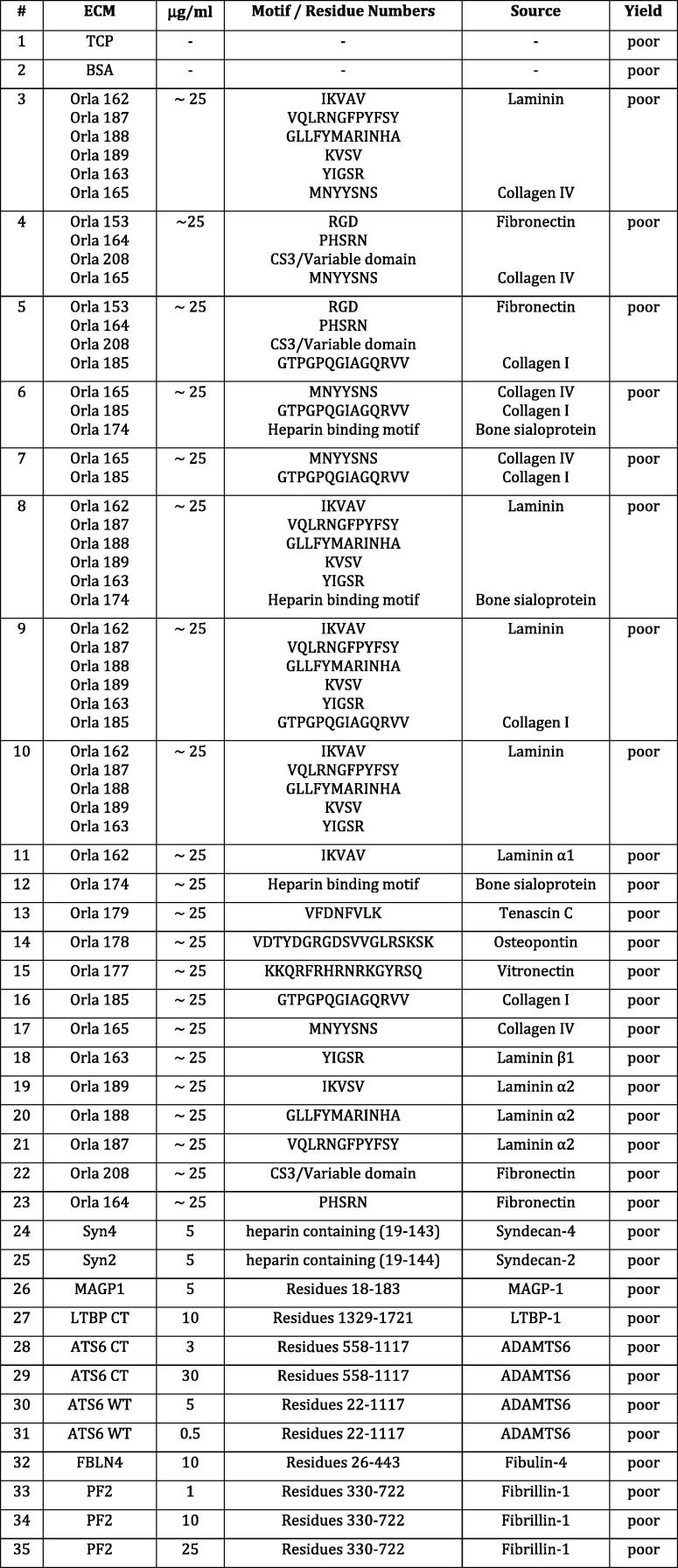

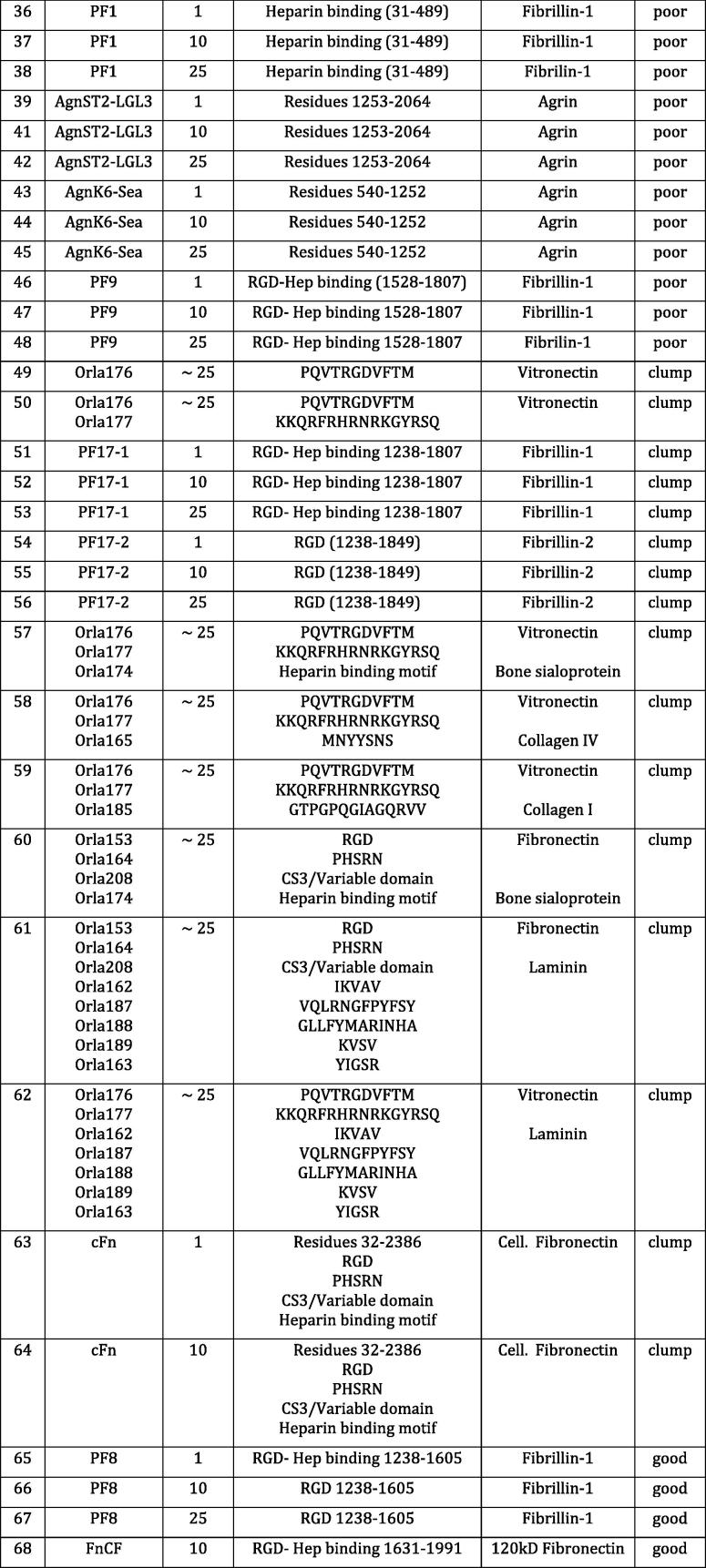

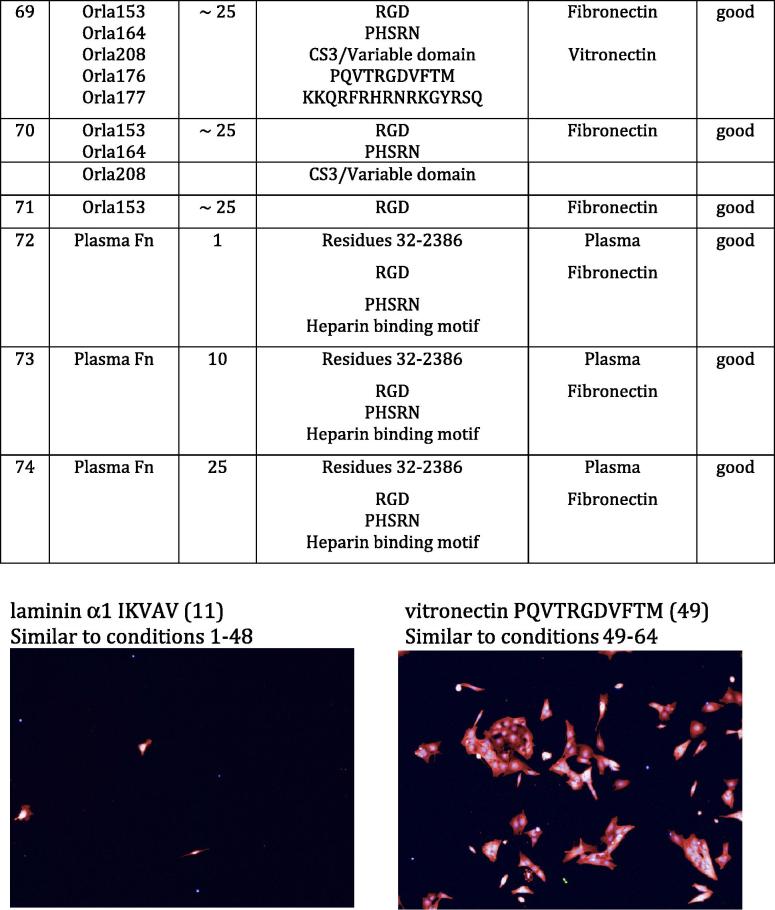
A screen of 74 conditions to visualise single iPS cells. List of tested substrate conditions from two combined arrays (Orla and custom made). Columns indicate an arbitrary reference number, name concentration, motifs or residues and source are detailed. Yield refers to number of single cells observed by visual inspection indicative of assay quality. The insets show examples of suboptimal substrates for iPSCs. Very few cells attach when plated on laminin α1 IKVAV (Table 1, n. 11, representative of conditions 1–48) and many clumps and very few single cells are observed when cells are plated on vitronectin PQVTRGDVFTM (Table 1, n. 49, representative of conditions 49–64).
